# Beyond Glucocorticoids: Integrating Dehydroepiandrosterone (DHEA) into Animal Welfare Research

**DOI:** 10.3390/ani10081381

**Published:** 2020-08-09

**Authors:** Jessica C. Whitham, Jocelyn L. Bryant, Lance J. Miller

**Affiliations:** 1 Chicago Zoological Society-Brookfield Zoo, 3300 Golf Road, Brookfield, IL 60513, USA; lance.miller@czs.org; 2 La Grange Highlands, IL 60525, USA; jocbryant@yahoo.com

**Keywords:** dehydroepiandrosterone, DHEA, dehydroepiandrosterone sulfate, glucocorticoids, cortisol, ratio, animal welfare

## Abstract

**Simple Summary:**

It is considered best practice in the field of animal welfare to utilize multiple indicators of welfare when assessing an individual’s current state. While welfare scientists have traditionally relied on glucocorticoids to gain insight into an animal’s physiological condition, there are limitations to relying solely on these biomarkers. Fortunately, other biomarkers, such as dehydroepiandrosterone and its sulfate ester—collectively referred to as DHEA(S)—help provide a more complete picture of hypothalamic–pituitary–adrenal (HPA) axis activity and functionality. There is evidence that the ratio of glucocorticoids:DHEA(S) may serve as an indicator of immune function, mental health, cognitive performance and overall welfare. The current review highlights studies that have utilized the ratio of glucocorticoids:DHEA(S), outlines methodological considerations and discusses how the ratio can best be applied to assess animal welfare.

**Abstract:**

Animal welfare researchers are committed to identifying novel measures for enhancing the quality of life of individual animals. Recently, welfare scientists have emphasized the need for tracking multiple indicators of an animal’s behavioral, emotional and mental health. Researchers are currently focused on integrating non-invasive physiological biomarkers to gain insight into an individual’s welfare status. Most commonly, the animal welfare community has analyzed glucocorticoid hormones and their metabolites as a measure of stress. While glucocorticoids provide valuable information about hypothalamic–pituitary–adrenal (HPA) axis activity, there are limitations to utilizing these hormones as the sole measure of long-term stress and welfare. Other biomarkers, such as dehydroepiandrosterone and its sulfate ester—collectively referred to as DHEA(S)—help provide a more complete picture of HPA activity. DHEA(S) counteracts the effects glucocorticoids by having anti-aging, immune-enhancing and neuroprotective properties. Recent studies have examined the ratio of glucocorticoids to DHEA(S) as a way to better understand how the HPA axis is functioning. There is evidence that this ratio serves as an indicator of immune function, mental health, cognitive performance and overall welfare. We review studies that employed the glucocorticoid:DHEA(S) ratio, outline methodological considerations and discuss how researchers can integrate glucocorticoids, DHEA(S) and the glucocorticoid:DHEA(S) ratio into welfare assessments.

## 1. Introduction

Animal welfare researchers continually strive to develop new methods for improving the quality of life of individual animals. Animal welfare is a concept that considers an animal’s physical, emotional, and mental states and is measured on a continuum from poor to good [1]. In recent years, animal care professionals working in managed settings have focused on identifying effective animal-based measures for systematically monitoring and assessing welfare [2,3]. Indeed, while the animal welfare community has traditionally conducted audits by evaluating specific management practices and environmental conditions, researchers now emphasize the importance of regularly tracking multiple indicators of behavioral, psychological and physiological health [2,3]. Furthermore, it is crucial that we focus on identifying measures that do not require invasive sampling or handling on a regular basis [3].

Recently, welfare scientists have increased efforts to non-invasively measure physiological biomarkers to gain insight into an animal’s physical condition, psychological health and overall welfare status [3,4]. Researchers are committed to incorporating biomarkers that not only reflect an animal’s level of arousal but, if possible, also the valence (positive or negative) of that arousal. These biomarkers include measures of heart rate, molecules linked to the sympathetic nervous system (e.g., alpha-amylase) and various proteins related to immune function (e.g., cytokines and immunoglobulin A) [4,5,6,7].

Most commonly, however, animal studies have focused on tracking glucocorticoid hormones (primarily cortisol for fish and most mammalian species; corticosterone for birds, reptiles, amphibians and rodents) or their metabolites as a measure of both long-term and short-term stress [8,9,10]. Due to the development of non-invasive monitoring techniques, glucocorticoids or their metabolites can be tracked in feces, urine, saliva, feathers, hair, fingernails, claws and skin secretions [9,10,11,12,13,14,15,16]. While glucocorticoids provide useful information about the activity of the hypothalamic–pituitary–adrenal (HPA) axis (or the hypothalamic–pituitary–interrenal axis for amphibians, fish and reptiles), there are limitations to tracking this measure alone [8,17,18]. Fortunately, recent research on human subjects indicates that incorporating other biomarkers of HPA activity, such as the “glucocorticoid antagonist” dehydroepiandrosterone (DHEA) and its sulfate ester (DHEA-S), may provide a more complete picture of how an individual’s HPA axis is functioning [19]. Moving forward, we will use DHEA(S) when referring to both DHEA and DHEA-S. 

In this review article, we provide an overview of the HPA axis, describe the primary actions of glucocorticoids and DHEA(S), discuss the pathophysiological effects of HPA dysfunction, and consider how the ratio of glucocorticoids to DHEA(S) may be applied to animal welfare research. However, we should note that while the cortisol:DHEA(S) ratio has been employed in studies of human development, psychology and immunology, there has been relatively little research on how this ratio may provide insight into the physical, mental and emotional health of non-human animals [15,19,20,21,22,23,24,25,26,27]. Therefore, we will explore the benefits of integrating this biomarker into animal welfare monitoring schemes and discuss potential methods for applying this metric in managed care settings. Finally, we should note that our discussion focuses on mammals, as few studies have investigated how DHEA(S) relates to welfare in other classes, and we want to avoid making generalizations about stress physiology, immunology and cognitive function in those taxonomic groups.

## 2. Materials and Methods 

To review the existing literature, we searched Articles Plus. We began our search with the following terms: dehydroepiandrosterone + animal welfare, DHEA + animal welfare, dehydroepiandrosterone sulfate + animal welfare, cortisol:dehydroepiandrosterone + animal welfare, glucocorticoid:dehydroepiandrosterone + animal welfare, cortisol:DHEA + animal welfare, and glucocorticoid:DHEA + animal welfare. We limited articles to those that were published in English and in peer-reviewed journals. This initial search resulted in 32 unique entries that were thoroughly read for suitability. Additional sources were discovered by searching these articles for other potential references. Ultimately, 195 articles were included in the review, including research articles and literature reviews. These final sources are listed in the References section. 

## 3. Overview of the Hypothalamic–Pituitary–Adrenal (HPA) Axis

**Figure 1:**
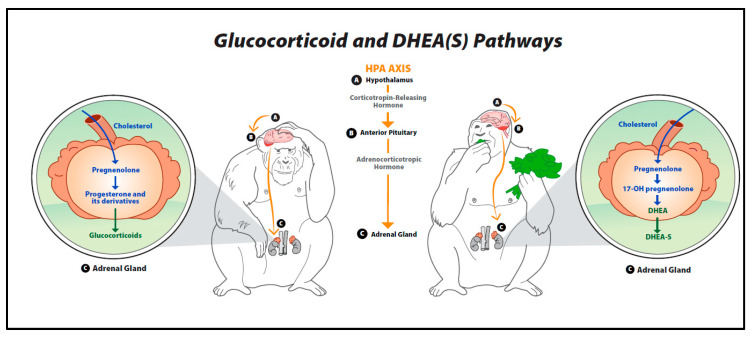
Glucocorticoid and dehydroepiandrosterone pathways.

The hypothalamic–pituitary–adrenal (HPA) axis is a hormonal response system that is crucial for maintaining a basal homeostatic state [17,19]. The hormonal cascade associated with this system can be activated by intrinsic or extrinsic events, including physical, emotional, and mental stressors [12,19]. Furthermore, these stressors may be either real or perceived [19]. Once the axis has been triggered by a stressor, the hypothalamus produces corticotropin-releasing hormone (CRH), which leads to the secretion of adrenocorticotropic hormone (ACTH) by the anterior pituitary gland. ACTH, in turn, stimulates the conversion of cholesterol to pregnenolone—a precursor to all steroid hormones including glucocorticoids, DHEA, progesterone, testosterone and estrogens [19,28,29,30]. These pathways are illustrated in Figure 1. Both DHEA and glucocorticoids are excreted mainly by the zona reticularis of the adrenal cortex [29]. If the HPA axis is functioning properly, a negative feedback loop inhibits the release of additional glucocorticoids after the stressor has diminished or passed [8,31]. Previous studies report that DHEA(S) does not exert feedback on the HPA axis [25,29]. As discussed in more detail below, both glucocorticoids and DHEA(S) have widespread effects on behavioral, emotional and physical health by impacting systems related to motivation, cognitive function, mood, immunologic function and sensory processing [25].

This figure provides an overview of the biosynthesis of glucocorticoids, dehydroepiandrosterone (DHEA) and DHEA’s sulfate ester (DHEA-S). Once the hypothalamic–pituitary–adrenal (HPA) axis is triggered by a stressor, the hypothalamus secretes corticotropin-releasing hormone (CRH), leading to the production of adrenocorticotropic hormone (ACTH) by the anterior pituitary gland. ACTH then stimulates the conversion of cholesterol to pregnenolone in the adrenal glands. Pregnenolone is a precursor to steroid hormones such as glucocorticoids (cortisol, corticosterone), DHEA, and DHEA-S. 

Exposure to repeated or chronic stressors can lead to the dysregulation of the HPA axis, resulting in pathophysiological effects [8,19,25,31,32,33]. Dysfunction of the HPA axis occurs when glucocorticoids are upregulated and DHEA(S) production is reduced [19]. This dysregulation—which will be discussed in more detail below—may disrupt various homeostatic mechanisms, suppress the immune system, and inhibit the production of growth and reproductive hormones, e.g., [8,18,19,25,33]. 

## 4. Glucocorticoids

Glucocorticoids are essential for survival, playing an important role in actions that involve the metabolic, immune and central nervous systems [17,31]. These steroid hormones are responsible for maintaining homeostasis by coordinating physiological responses to stress, exertion and arousal. Specifically, an increase in glucocorticoid production results in the secretion of glucose—providing the energy needed to overcome immediate challenges and restore homeostasis—while inhibiting processes associated with non-essential functions (e.g., growth) [19,25]. It is crucial to note that while acute stress—triggered by events such as mating, hunting and courtship—may be beneficial to an animal, repeated exposure to acute stressors or chronic activation of the HPA axis can lead to dysfunction. Indeed, while the HPA axis may initially be over-responsive, leading to hypercortisolism, hypocortisolism or “adrenal fatigue” may ultimately occur [18,31,32,33]. 

If an animal is chronically or repeatedly exposed to stressors, elevated levels of glucocorticoids may not be effectively downregulated by the negative feedback loop, resulting in hypercortisolism [19]. Hypercortisolism leads to additional HPA axis dysfunction, having damaging effects on cognition, immune function and mental health [18,25]. Elevated glucocorticoids even inhibit the release of the hormones needed to produce the anabolic steroids required for growth, healing and reproduction [19]. Research on both humans and other mammals has demonstrated that prolonged glucocorticoid elevation is associated with impairments in cognitive performance, including learning and memory tasks [28,34]. In fact, de Kloet and colleagues [28] note that chronically stressed animals not only exhibit major deficits in hippocampus-related memory but also an increase in fear-motivated behavior, with structural and cellular changes being documented in the hippocampus, amygdala and prefrontal cortex. Further, while glucocorticoids have anti-inflammatory actions when the HPA axis is functioning properly, overproduction can negatively impact the immune system in numerous ways [18,19,30]. For instance, hypercortisolism can reduce the production of certain lymphocytes, cytokines and antibodies, resulting in both an increased risk of developing infections and a diminished ability to recover and heal [25,30,35,36]. In terms of reproduction, prolonged exposure to high levels of glucocorticoids can affect the release of reproductive hormones, sexual receptivity and reproductive behavior [8,18,19,31]. Finally, hypercortisolism is linked to several mental health issues in humans [19,25,36,37,38]. Similarly, animal studies have reported a positive relationship between glucocorticoid concentrations and the occurrence of abnormal, repetitive and self-injurious behaviors [3,16,18]. For instance, Wielebnowski and colleagues [16] found that for clouded leopards living in North American zoos, mean fecal glucocorticoid metabolite concentrations were significantly correlated with behavioral issues, such as pacing, hiding and fur-plucking. In a separate article, Wielebnowski [18] reviews the pathologic symptoms associated with chronically elevated glucocorticoids (e.g., growth reduction, reproductive problems, muscle wasting, immune deficiency, and impaired neurological function) and discusses how animal welfare scientists can attempt to distinguish between stress and distress by using physiological, behavioral and health measures, see also [3]. Clearly, hypercortisolism can have devastating effects on an individual’s physical, mental and emotional well-being. 

Hypocortisolism can also negatively affect an individual’s health. When an animal is exposed to chronic or repeated stressors, an adaptation may occur within the HPA axis to protect the individual from chronically elevated glucocorticoids that threaten long-term survival [19]. This adaptation functions via reduced glucocorticoid signaling and alterations in the negative feedback loop, with upstream changes in CRH and ACTH resulting in reduced glucocorticoid production [25]. In fact, changes in several “levels” of the HPA axis may contribute to hypocortisolism [32]. Heim and colleagues [33] outline various mechanisms that may underlie the development and persistence of hypocortisolism, including reduced biosynthesis/depletion of certain hormones (e.g., CRH and ACTH), downregulation of pituitary corticotropin-releasing factor (CRF) receptors, increased feedback sensitivity, and morphological changes (e.g., atrophy of the hippocampus or adrenal gland). Reduced glucocorticoid secretion is associated with numerous health issues including an increased risk of developing inflammatory diseases, a heightened susceptibility to certain pathogens (e.g., parasites, allergens, and toxins), impaired cognitive function, and mental health or behavioral issues [19,30,33]. Indeed, there is evidence that chronic stress can lead to hypocortisolism and negative behaviors indicative of compromised welfare in various mammalian species [33,39,40]. For instance, in an experimental study, growing pigs housed in a poor environment exhibited lower baseline cortisol concentrations than those housed in an enriched environment (i.e., larger pens lined with straw bedding) and were more likely to direct manipulative social behaviors (e.g., biting and nosing) to penmates [39]. While a blunted glucocorticoid response may initially be adaptive for animals who regularly encounter stressors, hypocortisolism can be just as damaging as hypercortisolism. 

Several researchers have described the challenges and limitations of using glucocorticoids as a measure of stress or welfare [8,12,18]. It is crucial to remember that the HPA axis may be repeatedly activated by events or situations that are beneficial and do not negatively impact welfare over the long-term, such as stressors that occur during the breeding season [3,18]. In fact, HPA activation even occurs in response to positive events, such as exercise [3]. Therefore, it can be difficult to differentiate between an adaptive stress response and chronic stress or distress [18]. Further, as discussed above, both increases and decreases in baseline glucocorticoid concentrations can be associated with health issues and poor welfare [8,17]. Indeed, after conducting a comprehensive literature review and analysis, Dickens and Romero concluded that a generalized endocrine profile does not exist for wild animals facing chronic stress. The authors explain that glucocorticoid concentrations, “can result from changes in the negative feedback system, as a result of changes in perception of a stressor (responding versus not responding to a stimulus), or as a result of how the stressor signal travels through the secretory pathway” [8] (p. 181). Furthermore, a meta-analysis of the human literature determined that HPA activity is influenced by factors such as: time that has elapsed since exposure to a stressor, the nature of the stressor (e.g., a physical threat vs. social stress) and the controllability of the stressor [17]. This means that features related to both the stressor and the individual may impact HPA function, resulting in different hormonal profiles. 

Glucocorticoid concentrations may be influenced by a variety of other factors including the time of day, season, age, sex and reproductive condition [12,41]. For instance, a circadian rhythm of baseline glucocorticoid concentrations is found for most species [42,43]. For humans, cortisol increases significantly during awakening, peaks shortly thereafter, and then steadily declines throughout the day so that the lowest levels are detected in the evening [42]. Age-related changes in glucocorticoids are also widely reported [44,45]. In fact, there is evidence that hypercortisolism may be a feature of the aging process for many species [46]. See [44] for a thorough discussion of the “glucocorticoid cascade hypothesis”. It should be noted that this pattern does not hold for all species. For example, when considering adult male killer whales, those considered “aged” (at least 31 years old) had lower concentrations of glucocorticoids than their younger counterparts [47]. Finally, when integrating glucocorticoids into welfare studies, researchers should recognize that, as with other biomarkers, results may vary based on methodological differences related to sample type, data collection protocol, sample processing and analyses [10,12]. Methodological considerations and limitations related to the use of glucocorticoids in welfare research will be discussed in more detail below.

## 5. Dehydroepiandrosterone (DHEA)

Dehydroepiandrosterone (DHEA) and its sulfate ester (DHEA-S) have been characterized as glucocorticoid antagonists, immunostimulants, biomarkers of aging, and neuroprotective hormones [19,25,35,48]. DHEA and DHEA-S are the most abundant hormones produced by the adrenal glands in primates and serve as precursors to both androgens in males and active estrogens in females [29,48]. The metabolism of DHEA(S) to sex steroids occurs in several tissues and organs, including the liver, gonads, adrenals and peripheral tissues [29]. In addition, DHEA(S) binds to steroid hormone receptors and binds to, activates, and modulates the levels of nuclear receptors. It is important to note that in humans, 99% of circulating DHEA is the sulfate form [29]. 

When integrating DHEA(S) into research—and particularly welfare studies—it is crucial to recognize that DHEA(S) can originate from sources other than the adrenal glands. While all DHEA(S) is secreted by the adrenal glands in women, in men up to 25% of DHEA and 5% of DHEA-S is secreted by the testes [29]. DHEA(S) circulates at detectable levels in a variety of species, including rabbits, dogs, pigs, sheep, horses, and birds, though for some species these hormones may primarily (or only) originate from the gonads rather than the adrenal glands [49,50,51]. Rats and mice have extremely low or even undetectable concentrations of circulating DHEA(S), with little to no adrenal production [49,52]. Alternatively, for hamsters, adrenalectomy significantly decreases plasma DHEA levels, but castration does not [49]. Finally, it should be noted that for some species, DHEA(S) can also be synthesized de novo in the brain [53]. Clearly, the relative contribution of these organs and glands to DHEA(S) production must be considered before this biomarker can be applied to animal welfare research.

For certain species, DHEA(S) has widespread physiological effects that impact cognition, immune function and mental health, as well as behaviors related to survival and reproduction [25,35,54]. When DHEA is administered exogenously, it “protects” the hippocampus by counteracting the neurotoxic effects of glucocorticoids [55]. In fact, adult male humans participating in episodic memory tests showed improved recollection following the administration of DHEA [56]. Similarly, DHEA-S injections had memory enhancing effects in aging mice [57], and the administration of both DHEA and DHEA-S improved the cognitive performance of rats [58]. Frye and Lacey [58] suggested that DHEA(S) may impact cognitive performance by influencing a rat’s affective state (e.g., by reducing performance anxiety). In humans, maintaining appropriate levels of DHEA(S) is also associated with indicators of good mental health, including positive mood and reduced anxiety [25,56]. 

Recent research on DHEA(S) has also focused on its immune-enhancing and anti-aging properties [35]. DHEA increases the production of cytokines that promote white blood cell activity, while also inhibiting the production of cytokines responsible for inflammation [59,60,61]. This is consistent with Almeida and colleagues’ [20] findings that lame cows had 23% lower DHEA concentrations than healthy cows, but see [62]. In terms of DHEA(S)’s role in the aging process, Bauer [35] discusses how low DHEA(S) levels may contribute to immunosenescence and explains that replacement therapy can lead to improvements in memory, immune function and overall well-being [63]. There is even evidence that DHEA and DHEA-S protect against various types of mortality [64]. For example, in mice, DHEA administration resulted in improved immune function and even the increased likelihood of survival following trauma-hemorrhage (i.e., a laparotomy and hemorrhagic shock) and sepsis [65]; see also [66,67].

Finally, in non-human animals, DHEA(S) may play an important role in promoting species-appropriate or adaptive behaviors. For some species, DHEA(S) helps to modulate aggression by allowing, “… an individual to maintain aggression during specific life history stages without incurring the ‘costs’ of high circulating testosterone…” [51] (p. 485). DHEA also plays a role in regulating the territorial behavior of red squirrels during both the breeding season and non-breeding season [68].

While DHEA(S) may increase in response to acute stressors [69] and even serve a protective role by antagonizing the effects of cortisol [70,71], dysregulation of the HPA axis due to repeated or chronic stressors can lead to a reduction in DHEA(S) [19,25]. Indeed, Edes and colleagues [72] recommend incorporating DHEA-S into allostatic load indices for western lowland gorillas, as low levels of DHEA-S reflect physiological dysregulation. Such indices can be used to predict morbidity and mortality risk. In humans, suboptimal levels of DHEA(S) are reported for those suffering from chronic diseases, such as mood disorders, chronic pain disorders (e.g., fibromyalgia) and inflammatory diseases (e.g., inflammatory bowel disease) [19]. A more thorough review of how low levels of DHEA(S) may negatively impact human health can be read in detail elsewhere [19,25]. 

When integrating DHEA(S) into studies of stress and welfare, it is important to be cognizant of various limitations and challenges. For instance, although diurnal rhythms have been reported for DHEA and DHEA-S, species may exhibit wildly different patterns. For example, while DHEA and DHEA-S peak in the evening for golden hamsters, which are nocturnal [49], DHEA circulates at its highest levels in the morning for humans [73]. Interestingly, due to its slow rate of metabolic clearance and long half-life, DHEA-S does not have a strong diurnal rhythm or vary greatly from one day to the next in humans [29]. As a result, Kamin and Kertes argue that, for humans, DHEA-S may, “… represent a more stable index of adrenocortical activity and stress accumulated over time,” while, “DHEA may better reflect the response to stress acutely experienced” [25] (p. 72). It is also important to note that age-related changes in DHEA and DHEA-S have been reported for various species [48,74]. For killer whales, pubertal and adult males exhibit higher DHEA concentrations than juvenile males [47]. In humans, DHEA(S) levels change throughout development, peaking sometime between 20–30 years of age, and then drastically declining in the elderly [25,29]. Similarly, studies of various non-human primate species have reported that circulating DHEA and/or DHEA-S levels are lower in late adulthood than in young adulthood or for juveniles [48,75,76,77,78]. Sex differences in DHEA(S) levels may also exist for certain species. For example, Rosado and colleagues [50] reported that as compared to female dogs, male dogs exhibited significantly higher plasma DHEA concentrations. On the contrary, Pieper and Lobocki [49] found that while mean serum DHEA levels were higher in female hamsters, mean serum DHEA-S was higher in males. It is crucial to consider how the metabolism of DHEA(S) into sex steroids, and how this varies by sex and age, may confound the results of welfare studies. Clearly, welfare researchers must take a myriad of factors into consideration before initiating studies that include DHEA and DHEA-S as biomarkers [79]. 

## 6. Glucocorticoid:DHEA Ratio

There has been growing interest in examining the ratio of glucocorticoids to DHEA(S) to gain insight into how the HPA axis is functioning [19,80,81]. Kamin and Kertes describe the “antagonistic dynamic” between cortisol and DHEA(S) and explain that because they, “… mediate largely opposing biologic, neurologic, and immunologic functions… measuring their levels simultaneously may be an important indicator of net glucocorticoid activity” [25] (p. 72). In fact, although it may not be possible to identify HPA axis dysfunction by examining glucocorticoid levels alone, glucocorticoid:DHEA(S) ratios may be helpful [19,82,83,84]. For example, while those serving as caregivers for Alzheimer’s patients had similar cortisol levels as age-matched controls, the former had significantly lower DHEA-S levels and higher cortisol:DHEA-S ratios [85]. Furthermore, caregivers reported more symptoms of stress, anxiety and depression than controls, and were more likely to exhibit an impaired HPA axis response following the administration of a synthetic glucocorticoid. When chronic or repeated stressors disrupt the sensitive balance between glucocorticoids and DHEA(S), there may be detrimental effects to an individual’s physical, mental and emotional health [25].

There is convincing evidence that the cortisol:DHEA(S) ratio may serve as a robust indicator of immune function. A high cortisol:DHEA(S) ratio has been reported for humans suffering from severe injuries and illnesses, and may even be used to predict the risk of infection or death [64,86,87]. Phillips and colleagues’ [64] study of Vietnam veterans found that the cortisol:DHEA-S ratio was positively associated with various types of mortality over the next 15 years. In a study of patients suffering from septic shock and multiple trauma, the lowest DHEA-S levels and highest cortisol levels were found for the most critically ill individuals [86]. As a result, the authors suggest that the cortisol:DHEA-S ratio may serve as a prognostic indicator of the outcome of septic shock and other severe illnesses. It should be noted that age may also play a major factor in recovery, as Butcher and colleagues [87] found that elderly hip fracture patients had a higher cortisol:DHEA-S ratio than both age-matched controls and young hip fracture patients. The authors suggest that aging results in a more exaggerated response to traumatic injury, and that these increases in cortisol cannot be offset by high DHEA-S levels, as occurs with younger patients. Bauer argues that—even for healthy elders—peripheral tissues may be “vulnerable” to the actions of glucocorticoids due to “low protective DHEA levels” [35] (p. 242). In fact, he suggests that age-related increases in the cortisol:DHEA(S) ratio, “could be understood as a major determinant of immunological changes observed during aging” (p. 241). In other words, a high cortisol:DHEA(S) ratio may play a large role in immunosenescence [88].

Cognitive function and mental health may also be negatively impacted by high cortisol:DHEA(S) ratios. In Kalmijn and colleagues’ [89] study of elderly subjects, those with high cortisol:DHEA-S ratios were more likely to display cognitive impairment. Ferrari and colleagues [82] discovered that elderly subjects exhibited higher cortisol:DHEA-S ratios than young controls, and that the highest ratios were found for older individuals with dementia. Various studies on humans have demonstrated that elevated cortisol:DHEA(S) ratios are associated with treatment-resistant depression, anxiety, stressful life events, negative mood, schizophrenia, angry temperament, hostility and symptoms of dissociation [90,91,92,93,94]. For example, in a study of older men, a high morning cortisol:DHEA ratio was associated with greater anxiety, general mood disturbance, higher negative mood in the evening, confusion and lower episodic memory performance [95]. There is even evidence that a high cortisol:DHEA ratio can serve as a predictor of persistent major depression for youth presenting with major depression [83,96]. However, while a high cortisol:DHEA(S) ratio is often reported for youth with internalizing disorders, those with externalizing disorders may have a relatively low cortisol:DHEA(S) ratio [97]. Kamin and Kertes caution that, “rather than assuming that a low level of cortisol and a high level DHEA(S) are optimal, it is likely that both hormones need to be maintained at certain levels depending on biological and psychological states” [25] (p. 77). In other words, these hormones counterbalance one another and appropriate levels of each must be maintained.

Relatively fewer studies have specifically examined the ratio of glucocorticoids to DHEA(S) in other mammals and how this ratio relates to welfare. Goncharova and colleagues [23] determined that among old female rhesus macaques, those who exhibited “depression-like” behavior had significantly higher ratios than those classified as having “aggressive” or “average” behavior. The glucocorticoid:DHEA(S) ratio may also serve as a diagnostic or prognostic tool for some species in terms of physical health. For example, there is evidence that—as compared to healthy Holstein cows—lame cows had elevated cortisol:DHEA ratios (65% higher), had lower serum DHEA, exhibited less eating and ruminating, and performed more self-grooming [20]. As a result, Almeida and colleagues [20] suggested that the cortisol:DHEA ratio may serve as a biomarker of inflammatory foot lesions. Similarly, in a study of seals (harbor and gray), wild seals suffering from disease had lower serum DHEA concentrations and higher cortisol:DHEA ratios than both healthy wild seals and zoo-living seals [24]. Because serum cortisol did not differ between the wild healthy seals and wild diseased seals, the authors suggest that the cortisol:DHEA ratio may better reflect the functionality of the HPA axis.

A handful of studies have examined how transport, environmental conditions, housing and husbandry practices impact the glucocorticoid:DHEA ratio. An elevated cortisol:DHEA ratio was reported for pigs coping with a novel environment after being transported to a new facility [15]. Similarly, transportation stress was associated with a significant increase in cortisol:DHEA ratios in young bulls, due to elevated blood cortisol and a decrease in DHEA [21]. In terms of environmental conditions, Peric and colleagues’ [26] experimental study determined that deteriorating conditions led to an increase in the cortisol:DHEA ratio in dairy cows. In an experimental study of piglets, those housed under conditions aimed to enhance welfare (e.g., access to outdoor areas and no tail docking) exhibited higher salivary DHEA, lower salivary cortisol and lower cortisol:DHEA ratios than piglets reared in housing systems with more stressors (e.g., no outdoor access and castration without anesthesia) [22]. Finally, as compared to horses exposed to a traditional stable management style, those that experienced natural boarding practices had significantly lower cortisol:DHEA ratios and cortisol concentrations, as well as significantly higher DHEA concentrations [27]. Not surprisingly, several farm animal welfare researchers have referred to the cortisol:DHEA ratio as one potential biomarker of resilience and allostatic load [15,26,98].

As with glucocorticoids and DHEA(S), several considerations must be taken into account when applying this ratio to welfare research, including the subjects’ age and sex [23,50]. Similar to the findings from research on human subjects, studies of various primate species have demonstrated that aging is associated with increased cortisol:DHEA-S ratios [23,78,82,99,100]. Goncharova and colleagues argue that the age-related increase in the cortisol:DHEA ratio, “… is invariably associated with impairment in DHEA-S-mediated antiglucocorticoid activity, and consequently in enhanced glucocorticoid neurotoxicity” [23] (p. 861). Aside from the effects of aging, sex differences may also exist for some species, as it does for humans. For example, gilts (i.e., young female pigs) had significantly higher cortisol:DHEA ratios than barrows (i.e., young castrated males), and male dogs had significantly higher DHEA:cortisol ratios than female dogs—though it is important to recognize that the latter study reversed the ratio [50,98]. Further considerations for incorporating the glucocorticoid:DHEA ratio into welfare studies will be discussed in the next section.

## 7. General Methodological Considerations

Before embarking on a new research project, the investigator must consider a myriad of factors that will impact overall study design, sample type selection, data collection protocol, sample processing and data analyses [10,12]. For instance, the researcher should be aware of how results will be influenced by factors such as season, age, sex and reproductive condition [12,41]. For studies focused on assessing or monitoring welfare over the long-term, data collection may extend across several seasons and track the individual across various reproductive states. 

### 7.1. Diurnal Rhythms of Hormone Secretion

Before initiating a study, the researcher must determine whether diurnal patterns of secretory activity exist for glucocorticoids and/or DHEA(S) for the species of interest. As discussed above, diurnal patterns of glucocorticoid secretory activity have been reported for humans and non-human animals, but these patterns vary greatly across taxa [42,43]. It is vital that researchers take these daily cycles into account when determining what time samples should be collected, and sampling should remain consistent throughout the study period. 

Similarly, species-specific diurnal rhythms have been reported for DHEA and DHEA-S, with levels peaking in the morning for some species but in the evening for others [49,73]. Even though human DHEA-S shows a slight awakening response, it does not have strong diurnal cyclicity due to its long half-life [101,102]. For species that exhibit a slow metabolic clearance of DHEA-S, and therefore show little variation from one day to the next, this biomarker may serve as a better indicator of chronic stress than DHEA [25,29]. On the contrary, DHEA may better reflect acute stress responses for some species, as has been reported for humans [25,70]. The researcher should consider which biomarker is most appropriate for addressing their research questions. Furthermore, samples should be collected at roughly the same time each day. 

### 7.2. Seasonal Variation in Hormone Secretion

Seasonal variation also must be taken into account when designing welfare studies that incorporate glucocorticoids and DHEA(S). In humans, higher plasma and salivary cortisol levels were found in the winter months as compared to summer months [103]. In non-human animals, there can also be seasonal variation in glucocorticoids related to pre-hibernation changes in body mass [104], minimum ambient temperature [105] and breeding season [41]. For example, male tufted capuchins exhibit an increase in cortisol during the peak of adult female sexual activity [106]. 

Though less commonly studied, researchers have also reported seasonal differences in DHEA(S) secretion [51,68]. For example, a study of red squirrels found that plasma DHEA levels are elevated during the breeding season [68]. The authors note that while an ACTH treatment indicated that circulating DHEA was of adrenal origin, the gonads may also contribute to DHEA levels [68]. In male squirrel monkeys, serum DHEA levels peak during the breeding season, though in this case there is likely a significant testicular contribution [107]. Similarly, a study of male killer whales revealed that DHEA concentrations are higher in summer months than in the fall, with the source being largely gonadal rather than adrenal [47]. These studies highlight how vital it is to determine where DHEA(S) originates from—and the relative contribution of these sources—across the seasons for individuals of particular age-sex classes. 

Clearly, welfare researchers must consider how hormone production, and even metabolic demands, vary across the seasons [10]. After all, an animal’s metabolism may be impacted by its reproductive condition, so that metabolite excretion differs between the breeding season and non-breeding season [10]. Again, it is important to collect baseline data throughout the year and to control for seasonal effects whenever possible.

### 7.3. Subject-Related Factors Influencing Hormone Secretion 

The researcher must also consider how hormone levels may be impacted by other factors at the individual level, including the animal’s age, reproductive condition, social rank and overall health. As discussed in detail above, studies of humans and other mammals have revealed that glucocorticoid and DHEA(S) levels are affected by age [108,109]. There is also evidence that reproductive state influences glucocorticoid concentrations in a variety of species [110]. An animal’s social rank in the group may impact glucocorticoid production, as well [111]. While low-ranking individuals may exhibit higher glucocorticoid levels than dominant individuals in some species, the opposite has been found for other species [112,113]. In addition, Steyer and colleagues [114] introduced the latent state–trait (LST) theory, which suggests that individuals may differ based on early life experiences and personality traits. It is also important to remember that sex differences in DHEA(S) and glucocorticoid concentrations may exist for certain species [49,50,115]. Furthermore, the fact that DHEA(S) metabolizes into sex steroids—and that metabolism varies by sex and age—must be taken into account when utilizing DHEA(S) as a welfare biomarker. Sex differences should also be evaluated with a biological validation, which are described in more detail below [116].

### 7.4. Sample Types for Measuring Glucocorticoids and Dehydroepiandrosterone 

Selecting the appropriate sample type when measuring glucocorticoids and DHEA(S) requires investigators to consider their research question, duration of study, short vs. long term goals, feasibility of collection, safety, and the physiology and behavior of the study animal. Another factor to consider is whether it is preferred to have a point-in-time hormone value or a cumulative, pooled concentration. For instance, while blood samples provide a snapshot of total hormone concentration at one moment in time, it is also possible to measure hormone by-products (i.e., metabolites) in urine and fecal samples, though these offer a pooled concentration [116,117,118]. Fortunately, there is evidence that the amount of hormone in the bloodstream is proportional to the rate of metabolite excretion, therefore providing valid options for non-invasive sampling, which is preferred when conducting welfare research [116,117,118,119]. However, if metabolites are analyzed, the researcher must recognize that there are species-specific differences in the metabolites that are formed, the routes of metabolism, and the activity of bacterial enzymes involved in the conversion of steroid hormones [110].

Below, pertinent information is presented on each biological sample type, along with its benefits and challenges. Furthermore, Table 1 provides an overview of the main advantages and disadvantages associated with the most common samples types used in ex situ studies. 

#### 7.4.1. Blood Samples

For many vertebrates, blood has been the preferred sample type for measuring steroid hormones [118]. Glucocorticoid levels have been measured via blood in a wide array of species, including black bears [139], sea turtles [140], Magellanic penguins [141], brown treesnakes [142], mice [143] and various non-human primates [144]. Similarly, many non-human animal studies have used plasma or serum to measure DHEA(S), including research on hamsters [49], Old World Monkeys [48], domestic dogs [50] and dairy cows [20]. In order to collect blood, it is vital to consider the ease of collection, how quickly the sample can be collected and the safety of the researcher [118]. 

Plasma and serum measurement of hormones comes with several pros and cons. Using plasma or serum is more feasible in ex situ studies, as it is often challenging to obtain blood samples in the wild. However, blood collection is an invasive sampling technique and handling/capture-induced stress should be kept to a minimum, especially when investigating stress and welfare. Indeed, the collection process itself may cause an increase in glucocorticoids in as quickly as 2–5 min in birds [145], 20 min in manatees [146] and 30 min in felids [147]. On a positive note, the investigator is able to measure the hormone directly rather than measuring “broken down” metabolites. Further, as noted above, blood offers a point-in-time snapshot of the concentration of hormone, providing insight into the individual’s state at that moment [118].

When using blood to measure glucocorticoids and DHEA(S), it is important to distinguish between bound and unbound hormone. Bound hormone circulates in the bloodstream and tries to maintain equilibrium with the assistance of transport proteins [120]. Unbound “free” hormone has been taken out of circulation and is exerting action on a tissue. Unbound free hormone concentrations are of most value when assessing a stress response, as they show stimulation and patterns of adrenal activity [120]. A human blood sample consists of approximately 95% bound cortisol and DHEA(S), which is not biologically active and will not accurately reflect physiological activity [19]. Because both bound and unbound hormone concentration is present in a blood sample, it may be necessary to perform a calculation to obtain an estimate of free hormone concentration [121]. 

#### 7.4.2. Saliva Samples

Researchers have found less invasive techniques to examine glucocorticoids and DHEA(S), such as by analyzing saliva samples [122]. One major benefit to utilizing saliva is that it allows investigators to measure a free, unbound concentration of hormone [122]. Many studies on human subjects have used salivary measures of cortisol and DHEA(S) [95,102,128]. Saliva has also been the sample type of choice in many ex situ animal studies that measure cortisol [22,123], but is not commonly used in field studies due to difficulty of collection. Handling stress during collection has been shown to be less of a concern with saliva than with blood samples. For example, no capture-induced stress (i.e., significant increase in cortisol) was detected in domestic dogs for at least 4 min post-capture [124].

However, there are challenges associated with using oral fluid. Both the safety of the researcher and the subject’s willingness to cooperate must be taken into account. In humans, collection is simple and involves chewing on an absorbent material or salivating into a tube [118]. According to Gallagher and colleagues [128], salivary cortisol collected via both passive drool and a citric acid-treated salivette correlated highly with plasma cortisol levels, but only the samples collected via passive drool correlated with plasma DHEA levels. Another study on humans demonstrated that while using cotton-based absorbent material resulted in a significant correlation between salivary and plasma cortisol, no correlation was found between salivary and plasma DHEA [129]. It is crucial that investigators consider how different collection methods and sampling materials may impact the measurement of different steroid hormones.

The collection process for some other mammals is similar to that for humans. Most researchers implement techniques that encourage chewing, e.g., for rhesus monkeys [123] and shelter dogs [148]. Species that are easily trainable may willingly allow swabbing or offer passive drool to the investigator, e.g., pigs [22], Indian rhino [125], Asian elephant [126], and Great Apes [127]. Contamination should be kept to a minimum, as substances in the mouth prior to sampling (e.g., breast milk and blood) may influence results [131]. Salivary flow rate must also be examined, as differences in saliva production influence the amount of analyte measured. Therefore, one should record the volume of sample collected over a particular time period to calculate an output per unit of time [130]. Overall, saliva can be an effective sample type for measuring cortisol and DHEA(S), as long as optimal collection methods and materials are used for each hormone.

#### 7.4.3. Urine Samples

Another non-to-minimally invasive approach for gaining insight into HPA activity involves analyzing urine samples [116,118]. Use of this sample type is most effective for humans and trained animals, as it can be quite difficult to locate and collect in the field [118]. While urinary glucocorticoids and DHEA(S) are metabolized by the liver and kidneys, leaving only a small amount of native hormone [132], measurement of metabolites is common practice. In glucocorticoid excretion rate experiments on hares, the peak concentration of endogenous metabolites was detected after the first elimination of urine, while the peak concentration in feces occurred 1 day later [134]. Due to these species-specific time lags, excretion rates must be determined for the species of interest, and it is recommended that collection occur within the same 1 hr window each day [149].

Urinary measures of glucocorticoids and DHEA(S) have been examined in studies of both humans and non-human animals. For instance, in humans, measurement of urinary free cortisol has been the optimal choice for medical diagnoses such as kidney disease [150], hypertension [151] and Cushing’s disease [152]. Similarly, urine samples can be used to diagnose Cushing’s disease in non-human animals such as domestic dogs [153], gorillas [154] and domestic cats [155]. Welfare researchers have also used urine to assess adrenocortical activity in a wide variety of species, including frogs [156], elephants [157], domestic cats [158], domestic dogs [159], okapi [160] and gorillas [161]. In terms of DHEA(S), urinary DHEA has been analyzed to detect steroid abuse in humans [162] and to assess changes in DHEA and DHEA-S in relation to aging [163]. Urinary measures of DHEA-S have also been analyzed to create age-related hormonal profiles and to identify ontogenetic changes (e.g., the onset of adrenarche) in some Great Apes [133,164]. 

#### 7.4.4. Fecal Samples

Use of fecal hormone monitoring is rarely used in human research, as the same information can be found using more desirable sample types. In fact, no studies on fecal cortisol or DHEA(S) in humans were found while conducting the current literature search.

On the contrary, fecal samples are the most common non-invasive biological sample from which to measure glucocorticoids in animal welfare studies. This sample type can be collected without any disruption to the animal’s daily routine and does not require handling, resulting in no capture-induced stress [9,138]. As noted above, because steroid hormones undergo metabolization in the liver and kidneys prior to excretion, fecal samples—like urine samples—yield by-products, or metabolites, of cortisol, corticosterone and DHEA(S) [119,137]. However, it should be noted that some researchers have specifically analyzed the native hormone, such as studies that examined fecal cortisol levels in pied tamarins [135] and scimitar-horned oryx [136].

Before embarking on a fecal hormone study, the investigator must determine how long it takes for these metabolites to be excreted [116]. For instance, when considering glucocorticoids, there is a time lag from as little as one hour for animals that are small bodied or that defecate frequently, e.g., birds [165], to over 24 h for larger animals such as chimpanzees [132], as reviewed by [166]. Fortunately, a number of hormone assays can successfully measure fecal metabolite concentrations for a wide array of species—from rats to North Atlantic right whales to European stonechat birds [118,167,168]. However, as explained in more detail below, both biochemical validation and biological validation are key in determining the effectiveness of the assay.

There are some limitations and drawbacks to utilizing fecal samples. For example, the researcher must consider how much time has passed between defecation and collection, as exposure to the elements may impact bacterial metabolism and either increase or decrease metabolite concentrations [10,138]. Furthermore, for socially-housed animals, it may be necessary to add a marker (e.g., food coloring) to the subjects’ diets in order to distinguish samples [116]. 

Recently, Palme [116] published a thorough review of non-human animal studies that relied on fecal glucocorticoid metabolite analysis, identifying 1327 papers. Research investigating both glucocorticoid and DHEA(S) metabolites in feces is less common [116,169,170]. Because fecal hormone monitoring has become the method of choice for non-invasive sampling, our discussion of assay selection below will mainly focus on this approach. 

#### 7.4.5. Other Sample Types

Recently, investigators have identified novel methods for measuring glucocorticoids and DHEA(S). For instance, both biomarkers have been examined by analyzing hair samples in a variety of species. In humans, hair has a fairly consistent growth rate of approximately 1 cm/month, so each cm of hair would be representative of the most recent month [171]. Hair cortisol measurement has been effective in both human research [172] and studies of other mammals [173]. In fact, measuring hair cortisol and DHEA levels, as well as examining the ratio of these two hormones, has already been used to assess stress in humans [174,175], pigs [15,26,98] and horses [27]. 

Measurement of glucocorticoids and DHEA in fingernails or claws may also be an option for some species, as it was discovered that endogenous hormones become infused into keratin during nail formation in humans [176]. A pilot study performed by Warnock and colleagues [177], determined that cortisol and DHEA could be measured in human fingernails and even found that the cortisol:DHEA ratio increased during times of stress. Similarly, Baxter-Gilbert and colleagues [11] determined that turtle claw trimmings could be used to measure corticosterone and suggested that this technique be applied to assess chronic stress. Hair, fingernail, and claw samples offer a more long-term measure of the hormone of interest.

Other class-specific biological samples are currently being tested to assess their efficacy in measuring glucocorticoids. For example, feathers have been used to measure glucocorticoids in house sparrows and house finches [13,178]. Feather samples reflect steroid concentration at the time of feather growth and can provide a long-term measure [179]. Santymire and colleagues [14] discovered that, for a variety of amphibian species, skin secretions showed an increase in glucocorticoids following an acute stressor. Other novel techniques such as analyzing snake sheds [180] and water obtained from fish tanks [181] are being evaluated as potential methods for detecting changes in adrenal activity. 

## 8. Quantifying Hormones and Hormone Metabolites

### 8.1. Radio-Immunoassays and Enzyme-Immunoassays

In non-human animal studies, immunoassays are the most common method for measuring concentrations of glucocorticoids, DHEA(S) and their metabolites [116]. In brief, immunoassays are a random competitive binding technique in which the hormones from a sample compete with labelled hormone for limited antibody binding sites [182]. Radio-immunoassays (RIA) and enzyme-immunoassays (EIA) are the most commonly used immunoassays. The difference between RIAs and EIAs lies in the detection system used to quantify the concentration in each sample. RIAs use a radioactive isotope as a detection label, which generates a radioactive signal that can be measured by a gamma counter [126]. EIAs utilize an enzyme label that produces a colorimetric signal that is measured by a spectrophotometer [118]. When considering recent non-human animal studies that conducted fecal glucocorticoid metabolite analyses, EIAs have been employed more frequently than RIAs (870 vs 370 papers) [116]. This is due to the fact that EIAs do not require any special permits, are relatively safe to use, and involve less expensive equipment [117]. 

Immunoassays are highly sensitive to the hormone molecule of interest. While these assays were originally developed for analyzing blood samples, researchers have since applied them to other biological samples. Investigators must rely heavily on the cross-reactivities of their antibody, particularly for feces in which only metabolites of the native hormone remain [183]. This limitation inspired the creation of group-specific EIAs that can detect a variety of glucocorticoid metabolites [116].

There are various immunoassays that can be employed to measure glucocorticoids and DHEA(S). The cost of the antibody is often the main consideration when selecting an assay. Glucocorticoid EIA assays can be made in house at low cost [184]. There is also a glucocorticoid RIA assay that has been utilized by a lab that processes a large quantity of samples on a regular basis [185]. Though more expensive, commercial RIA and EIA kits for the measurement of glucocorticoids and DHEA(S) are commonly employed, as they are readily available and easy to implement. Over 1200 published animal studies have used a commercially available RIA or EIA kit to measure fecal glucocorticoid metabolites [116]. Similarly, many studies have used commercial RIA and EIA kits to measure DHEA or DHEA-S in a variety of sample types [108,137,170,177,186].

### 8.2. Assay Validation

Regardless of sample type and assay system selected, it is vital to validate each assay for each study species to ensure accurate measurement of glucocorticoids, DHEA(S) or their metabolites. As stated by Sheriff and colleagues [118], there are a handful of requirements for validating immunoassays. First, the cross-reactivity of the antibody must be known, and the researcher should be aware of any other steroids or metabolites that can be detected by the antibody [187]. At the same time, it is important that the investigator identify other steroids and metabolites that are present in the sample. This is especially important when conducting fecal hormone analyses, as bacterial enzymes in the gut further metabolize and convert steroids [116]. Radioinfusion studies, as well as high-pressure liquid chromatography (HPLC) combined with mass spectrometry (MS), have become useful tools for discovering the steroid hormone metabolites present in a sample [110,116]. If it is not feasible to apply these methods, it is possible to perform a hormone challenge—as described below—and evaluate various immunoassays and group-specific antibodies to determine which best quantify the metabolites of interest [116]. 

Next, the researcher must perform biochemical validations. The first is a parallelism that determines whether a linear decrease in sample concentrations occurs when they are serially diluted and run parallel to the standard curve [118]. Specifically, we recommend performing serial two-fold dilutions of a sample pool to test for parallel displacement, which allows us to evaluate the antibody’s binding capacity. A recovery test is also needed to demonstrate that the immunoassay is detecting endogenous hormone. If the sample type contains mainly metabolites of the native hormone—as with fecal samples—this test may be somewhat artificial, and the use of HPLC immunograms would be more informative [166]. Fortunately, some enzyme-linked immunosorbent assay (ELISA) test kits are successful at detecting native hormones [135]. Finally, a series of analytical validations must occur to provide evidence of assay precision and accuracy. One way to accomplish this involves monitoring an inter-assay coefficient of variation (CV), typically of a low and high control [116]. Additionally, an intra-assay CV is helpful to show variability within one assay.

A crucial step in the validation process involves performing a biological validation by eliciting a change in circulating hormones in a standardized, systematic way. A common method for inducing a peak in glucocorticoids is to perform an adrenocorticotropic hormone (ACTH) challenge [188]. After administration of an ACTH injection, the adrenal cortex releases glucocorticoids into circulation [116]. A similar peak in glucocorticoids may even occur in response to a saline injection, as the injection itself is often an acute stressor [189]. Other methods of inducing a stress response include handling, transport, and environmental changes [190]. There is also evidence that DHEA increases in response to an ACTH challenge [68] and after experiencing an acute stressor [191].

Lastly, researchers must consider other potential limitations and challenges. For instance, if the assay has a high inter- or intra-assay CV, false positives in blanks, or non-specific binding, it may be best to try a different immunoassay [118]. It is also important to note that even if researchers follow the methods of previous studies and employ the same antibody, inter-laboratory variation is possible [192].

### 8.3. Additional Physiological Validation for Dehydroepiandrosterone 

An additional physiological validation is recommended for DHEA(S). Due to the fact that DHEA(S) may originate from non-adrenal sources, it is necessary to determine how the gonads may contribute to DHEA(S) production for the species of interest. For example, for non-human primate species, researchers have successfully conducted a human chorionic gonadotropin (hCG) hormone challenge to determine the direct effect of luteinizing hormone (LH)/hCG on DHEA(S) production [193]. This physiological validation allows the investigator to identify any gonadal contribution to DHEA(S) levels for individuals of particular age-sex classes.

## 9. Conclusions

With mounting criticism surrounding the use of glucocorticoid “stress hormones” as the sole indicator of HPA activity, additional biomarkers are necessary to better assess the welfare of individual animals [8,17,18]. While the field of animal welfare science continues to grow, it is still years behind the field of human health and well-being [194]. There is an abundance of evidence demonstrating that the cortisol:DHEA ratio can serve as a robust indicator of chronic stress in humans [19,25]. There is also a growing body of research suggesting that the glucocorticoid:DHEA ratio may be a valuable indicator of long-term stress for various non-human animal species. Clearly, more research is necessary to better understand the use of this biomarker across diverse taxonomic groups.

Employing the glucocorticoid:DHEA ratio across diverse taxonomic groups could have a substantial impact throughout the animal welfare community. Welfare scientists working in laboratories, agricultural settings, companion animal shelters, conservation centers and zoological facilities would have a novel tool for assessing whether an animal is in a positive, physiologically healthy state versus an impaired state that may reflect the individual’s inability to cope with its current environment. This information would allow professional caregivers to make informed management decisions and carry out interventions designed to enhance welfare. As the lack of negative behaviors does not suggest that an individual animal is thriving, a multifaceted approach that includes valid physiological indicators, such as the glucocorticoid:DHEA ratio, could be extremely beneficial. 

Moving forward, researchers should attempt to validate the glucocorticoid:DHEA(S) ratio as an indicator of welfare across a variety of taxa. This would involve conducting both biochemical and biological validations for a wide array of species to better understand the generalizability of the ratio for future research. As discussed above, biochemical validations must demonstrate parallelism for assays and explore antibody cross-reactivity to ensure accurate results [4]. Biological validations should evaluate individuals when they experience both positive situations (e.g., access to enrichment) and negative situations (e.g., veterinary procedures). It is also crucial to highlight sex differences and age-related differences in glucocorticoids and DHEA(S) to investigate how these hormones may fluctuate across seasons and to identify any diurnal patterns. Addressing these questions will help determine how the ratio varies across contexts, across life history stages, and in response to both acute and chronic stressors. Furthermore, for species with detectable levels of both DHEA and DHEA-S, the researcher should consider which biomarker is more appropriate for investigating acute versus chronic stress. For instance, it has been suggested that DHEA-S may be a better measure of long-term stress in humans, as it has a slower rate of metabolic clearance and longer half-life than DHEA [25]. It is critical that researchers determine species-specific metabolic clearance rates—as well as other aspects of metabolism and enzymatic activity—when refining research questions, designing their studies and choosing the most appropriate sample type. 

Combining the glucocorticoid:DHEA ratio with other indicators of welfare will be vital for thoroughly assessing the physical, mental and emotional health of individual animals. Examples of other valuable measures include behavioral data, results from cognitive bias tests and other physiological biomarkers of welfare (e.g., immunoglobulin A and cytokines). Adopting a multi-faceted approach would not only provide more insight into the current condition of the individual but would also help evaluate the usefulness of the ratio as an indicator of animal welfare [3].

While many species thrive within zoos and aquariums, others do not demonstrate species-appropriate behavioral profiles or reproduce successfully [195]. Having a wide variety of tools available for examining the welfare of animals under professional care not only benefits those individuals but can also inform future management decisions. The ability to identify negative situations, determine which conditions promote good welfare, and predict which individuals may be better at coping with certain events and environments allows for the continued improvement of care.

## Figures and Tables

**Table 1:** Common sample types for examining glucocorticoids and dehydroepiandrosterone.

Sample Type	Description	Main Advantages	Main Disadvantages
**Blood**	Offers point-in-time hormone value, providing insight into the subject’s current state [118].	Directly measures the hormone [118].	Invasive—may cause capture-induced stress [117,118,119]. May have to distinguish between bound and unbound, free hormone [120,121].
**Saliva**	Offers point-in-time hormone value similar to blood hormone concentrations [122].	Directly measures unbound, free concentration of the hormone [122]. Non-to-minimally invasive. Little disruption to the subject’s routine [22,123,124].	Subject must be trained and willing to cooperate [22,117,125,126,127]. Collection methods and sampling materials may impact measurement [128,129]. Must examine salivary flow rate [130]. Must avoid contamination from food [131].
**Urine**	It may be possible to detect the native hormone [132,133]. Metabolites offer a cumulative, pooled concentration [116,132].	Non-to-minimally invasive. Little disruption to the subject’s routine [116,118].	Requires either training the subject OR designing/modifying holding areas for sample collection [118]. Must determine excretion rates [134].
**Feces**	It may be possible to detect the native hormone [135,136]. Metabolites offer a cumulative, pooled concentration [119,137].	Non-invasive. No disruption to the subject’s routine [9,138].	May need to add a marker (e.g., food coloring) to distinguish the samples of socially-housed animals [116]. Must determine excretion rates [116].
